# Integrated analysis of breast cancer cell lines reveals unique signaling pathways

**DOI:** 10.1186/gb-2009-10-3-r31

**Published:** 2009-03-25

**Authors:** Laura M Heiser, Nicholas J Wang, Carolyn L Talcott, Keith R Laderoute, Merrill Knapp, Yinghui Guan, Zhi Hu, Safiyyah Ziyad, Barbara L Weber, Sylvie Laquerre, Jeffrey R Jackson, Richard F Wooster, Wen Lin Kuo, Joe W Gray, Paul T Spellman

**Affiliations:** 1 Life Sciences Division, Lawrence Berkeley National Laboratory, Cyclotron Rd., Berkeley, CA 94720, USA; 2 SRI International Inc., Ravenswood Ave, Menlo Park, CA 94025, USA; 3 Oncology CEDD, GlaxoSmithKline, Swedeland Rd, King of Prussia, PA 19406, USA; 4 Comprehensive Cancer Center, Sutter Street, University of California, San Francisco, CA 94143, USA

## Abstract

**Background:**

Cancer is a heterogeneous disease resulting from the accumulation of genetic defects that negatively impact control of cell division, motility, adhesion and apoptosis. Deregulation in signaling along the EgfR-MAPK pathway is common in breast cancer, though the manner in which deregulation occurs varies between both individuals and cancer subtypes.

**Results:**

We were interested in identifying subnetworks within the EgfR-MAPK pathway that are similarly deregulated across subsets of breast cancers. To that end, we mapped genomic, transcriptional and proteomic profiles for 30 breast cancer cell lines onto a curated Pathway Logic symbolic systems model of EgfR-MAPK signaling. This model was composed of 539 molecular states and 396 rules governing signaling between active states. We analyzed these models and identified several subtype-specific subnetworks, including one that suggested Pak1 is particularly important in regulating the MAPK cascade when it is over-expressed. We hypothesized that Pak1 over-expressing cell lines would have increased sensitivity to Mek inhibitors. We tested this experimentally by measuring quantitative responses of 20 breast cancer cell lines to three Mek inhibitors. We found that Pak1 over-expressing luminal breast cancer cell lines are significantly more sensitive to Mek inhibition compared to those that express Pak1 at low levels. This indicates that Pak1 over-expression may be a useful clinical marker to identify patient populations that may be sensitive to Mek inhibitors.

**Conclusions:**

All together, our results support the utility of symbolic system biology models for identification of therapeutic approaches that will be effective against breast cancer subsets.

## Abstract 2

Mapping of sub-networks in the EGFR-MAPK pathway in different breast cancer cell lines reveals that PAK1 may be a marker for sensitivity to MEK inhibitors.

## Background

Cancer is a heterogeneous disease that results from the accumulation of multiple genetic and epigenetic defects [1-4]. These defects lead to deregulation in cell signaling and, ultimately, impact control of cell division, motility, adhesion and apoptosis [5]. The mitogen-activated protein kinase (MAPK)/Erk pathway plays a central role in cell communication: it orchestrates signaling from external receptors to internal transcriptional machinery, which leads to changes in phenotype [6,7]. This pathway has been implicated in the origin of multiple carcinomas, including those of the breast [8-10]. Activation of MAPK is initiated by one of the four ErbB receptors (ErbB1/epidermal growth factor receptor (EgfR), ErbB2-4), which leads to signaling through Raf (RAF proto-oncogene serine/threonine-protein kinase), Mek (mitogen-activated protein kinase kinase 1/2) and Erk. In addition, the ErbB receptors integrate a diverse array of signals, both at the cell surface level and through cross-talk with other pathways, such as the phosphoinositide 3-kinase (Pi3k) pathway [11]. Both EgfR and ErbB2 are overexpressed in a substantial fraction of breast cancers and are recognized targets for breast cancer therapy [12-16]. In addition, Mek has long been studied as a therapeutic target, and many drugs that inhibit it are currently under development [17-20].

Among breast cancers, unique subsets can be defined at the genomic, transcriptional and proteomic levels. For many years, breast cancers were classified by whether or not they express various receptors, namely the estrogen receptor (ER/EsR1), the progesterone receptor (PR/PGR) and ErbB2 [21-25]. This key insight has been used to tailor therapies to individual patients [22,26]. Of particular interest is the finding that ER-negative tumors frequently show elevated signaling along the MAPK pathway compared to ER-positive cancers [27]. DNA amplification at various loci can also be used to stratify patients, and, importantly, has prognostic value as well [28,29]. For example, amplification at 8p12 and 17q12 are both associated with poor outcome [28,30]. The emergence of expression profiling technology led to the seminal observation that breast cancers can be systematically classified at the transcriptional level [23-25]. More recently, interest has turned toward the analysis of somatic mutations [31]. Different cancer types show common patterns of mutation, implying that a few key mutations play a pivotal role in tumorigenesis. All together, these studies indicate the value of identifying unique subsets of cancers, both for understanding the origin of the disease as well as identification of appropriate therapeutics.

A critical question remaining is how to identify meaningful subsets of cancers that differ in their cell signaling pathways. One approach to this problem is to identify gene expression signatures that reflect the activation status of oncogenic pathways [32,33]. While it is possible to stratify cancers into unique populations based on their expression patterns of these signatures, a key challenge lies in interpreting the meaning of the various genes within these signatures [34]. Here, we used an alternative approach in which we explored subtype-dependent behavior in genes that make up known signaling pathways.

Our goal was to identify signaling pathway modules that are deregulated in particular cancer subtypes. To that end, we populated a well-curated cell signaling model with molecular information from a panel of breast cancer cell lines. We used a combination of transcriptional, proteomic and mutational data to create a unique signaling network for each cell line. Specifically, we discretized transcript and protein data and used them to populate the network models; genes or proteins that are differentially expressed across the cell lines were evaluated as present in some cell lines and absent from others. The resultant network models can be viewed as a statistical formalism of the pathways activated in each of the cell lines.

**Figure 1:**
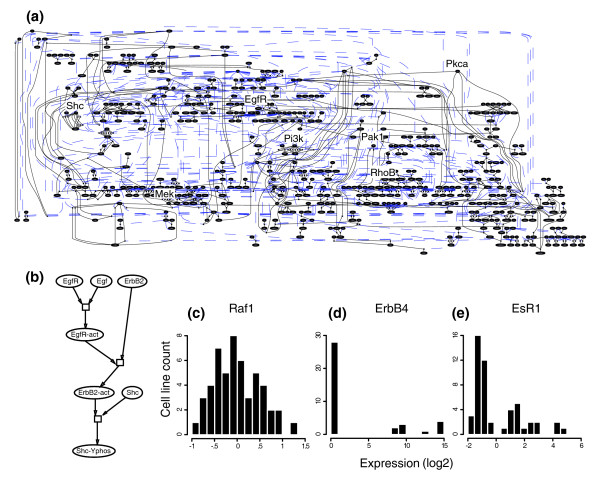
The signaling networks include several hundred components, all connected in a discrete manner. **(a) **Example network. Each circle represents a component in the network; lines represent connections between them (that is, rules). Key signaling components are noted. **(b) **A small subnetwork. **(c-e) **Examples of data used to populate the model. Each histogram shows the distribution of expression values across the complete panel of cell lines. Data for each component in the model were clustered individually to determine whether or not the component should be included in the initial state. Components that clustered into two groups were present in the initial states of some cell lines and absent from others. (c) Raf1 transcript data yields a single group. (d) ErbB4 protein data yields two groups. (e) EsR1 yields three groups.

We created our network models with Pathway Logic [35-38], a system designed to build discrete, logical (rule-based) models of signal transduction pathways [39]. Logical models are directly related to the canonical schematic diagrams ('cartoons') commonly used to show functional relationships among proteins, and, as such, are easily interpretable in the context of biological systems (Figure 1b) [40]. The two critical elements of a Pathway Logic model are a rule set and an initial state. The rules represent biochemical reactions, and the initial state is a representation of all proteins present in a particular cell line. Our model contains a rich rule set: the interactions between proteins have all been individually curated from primary literature sources and, therefore represent well-characterized signaling biology. In short, we used our collection of molecular data to identify active states in each cell line, and the rules to define signaling between these active states. The resultant networks are static coarse graphical representations of signaling that can be used to generate hypotheses about key signaling events in subsets of the cell lines.

We focused our modeling on the ErbB/MAPK pathway because deregulation along this pathway is both frequent in breast cancers and heterogeneous across them [12,41]. Further, it is involved in a complex web of signaling that results from cross-talk with other pathways [42]. Our model system includes rules that describe: interactions between the ErbB receptors and their ligands; direct association of intracellular signaling proteins with phosphorylated ErbB receptors; signaling along the canonical Raf-Mek-Erk pathway; cross-talk with Pi3k and Jak/Stat pathways; activation of immediate-early transcription factors (for example, Jun and Fos); and signaling from other receptors that influence MAPK signaling, including EphA2 (Ephrin type-A receptor 2 precursor) and integrins.

Our panel of cell lines captures many features of biological variation found in primary breast tumors [43]. Both the cell lines and tumors cluster into basal (EsR1-negative, Caveolin-1 (Cav1)-positive) and luminal (EsR1-positive, ErbB3-positive) expression subsets. These two subtypes - basal and luminal - also show distinct biological characteristics, including differences in morphology and invasive potential [23,25]. In addition, the cell lines show a broad response to pathway-targeted drugs (Gray *et al*., unpublished data). Overall, the genomic heterogeneity in the cell lines mirrors that observed in a large population of primary tumors, and as an ensemble constitutes a useful model of the molecular diversity of primary tumors [43].

We generated signaling network models for our panel of cell lines with the goal of identifying subnetworks that are active in particular subsets of cell lines. We found that the discretized data used to populate the initial states of the networks showed only a small amount of variation. Specifically, only 13% of the components in the initial state of the networks varied across the cell lines. Even with this small amount of variation, the discretized data used in the initial states could be clustered into basal and luminal cell line groups. Surprisingly, over half of the protein interactions predicted to occur varied across the cell line network models. In order to identify active subnetworks, we clustered the network features of our models, which resulted in three main groups of cell lines: basal, luminal and a third mixed group composed of both basal and luminal cell lines. In addition, we identified several network modules active in specific subsets of the cell lines. One module in particular implicated Pak1 (p21 protein (Cdc42/Rac)-activated kinase 1) as a key regulator of the Raf-Mek-Erk pathway in the subset of Pak1 over-expressing cell lines. We found that among luminal cell lines, the over-expression of Pak1 was significantly associated with sensitivity to Mek inhibition. Taken together, these results indicate that our modeling approach can be used to identify signaling subnetworks that are particularly important in subsets of breast cancer cell lines.

## Results

### Data clustering and model initialization

Our goal was to create a unique signaling network model for each cell line in our panel. In generating these models, we must accommodate two fundamental biological principles. First, the ErbB network results from the integration of many diverse signals, and second, most cell signaling occurs through protein-protein interactions. Ideally, then, we would create large networks populated with protein data. However, the acquisition of comprehensive protein abundance data for multiple cell lines is not technically feasible, so we used transcript data to infer protein levels when protein data were unavailable. An example of one of these large computed networks is shown in Figure 1a.

A key feature of Pathway Logic models is that they are discrete, so components are considered either present or absent. In order to populate our network models, we first discretized the transcript and protein data (see Materials and methods; Figure 1c-e). Following discretization, we determined which components (proteins) were present in the initial state of each cell line. We considered genes and proteins that are differentially expressed across the cell lines to be present in some cell lines and absent from others. Genes and proteins that showed little variation in expression were considered present in all cell lines. Although this approach is coarse, we can use it to assess which pathways may be most critical in each of the cell lines. That is, we can identify the pathways that may be highly up- or down-regulated in particular cell lines. This discretization algorithm captured many well-documented differences in expression across the cell lines. For example, the transcript data for EsR1 yields three clusters, which parallels the observation that primary breast tumors show varied expression of this protein (Figure 1e) [44,45].

The initial states were constructed from a population of 286 signaling components. We had expression data alone for 191 of these components, both protein and expression data for 25, and no available data for the 70 remaining components. Following discretization, 13 out of 25 (52%) proteins and 19 out of 191 (10%) transcripts form both present and absent groups. For the remaining protein and transcript data, a single group best describes the distribution of expression values. To explore the transcript and protein data further, we compared the clustering results for the 25 components that had both protein and transcript data available. Approximately two-thirds of these components show a high level of concordance between the two discretized datasets: nine yield a single present group for both datasets; eight yield a present and absent group for both datasets (mean Pearson's r = 0.603). The remaining eight components form a single group in one dataset and two groups in the other. For six of these, the transcript data yield a single group while the protein data form two groups (Table 1).

**Table 1:** Comparison of discretized protein and transcript data

	Protein clusters	Transcript clusters	Pearson's correlation
Irs1	2	2	0.0354
EgfR	2	2	0.491
ErbB3	2	2	0.491
Cav1	2	2	0.523
CD44	2	2	0.6
Cav2	2	2	0.882
EsR1	2	2	0.883
Cdh1	2	2	0.923
Akt1	1	1	-
Grb2	1	1	-
Hras	1	1	-
Igf1R	1	1	-
Jak1	1	1	-
Kras	1	1	-
MAPK1	1	1	-
MAPK3	1	1	-
Ptk2	1	1	-
ErbB2	1	2	-
Grb7	1	2	-
CtnnB1	2	1	-
Efna1	2	1	-
ErbB4	2	1	-
Rela	2	1	-
Src	2	1	-
Jun	2	1	-

We used the Sanger COSMIC database to identify mutations to Kras (Transforming protein p21 K-Ras 2/Ki-Ras/c-K-ras), Pten (Phosphatidylinositol-3,4,5-trisphosphate 3-phosphatase) and Pik3ca (PI3-kinase p110 subunit alpha) in our cell lines, and included these data in the initial states [46]. We focused on mutations in these three proteins for two reasons: first, they influence MAPK signaling, and second, the mutations have a known functional impact, so it is possible to computationally model them. Specifically, a G13D point mutation in Kras causes it to become constitutively active [47,48]. A frameshift mutation in Pten leads to premature termination and an inactive protein [49]. Three common point mutations in Pik3ca (E542K, E545K and H1047R) lead to increased lipid kinase activity [50,51]. Pik3ca is the most frequently mutated gene in our cell line panel (6 of 30; 20%), a finding that parallels other reports [52].

### Initial states reflect the known biology

**Figure 2:**
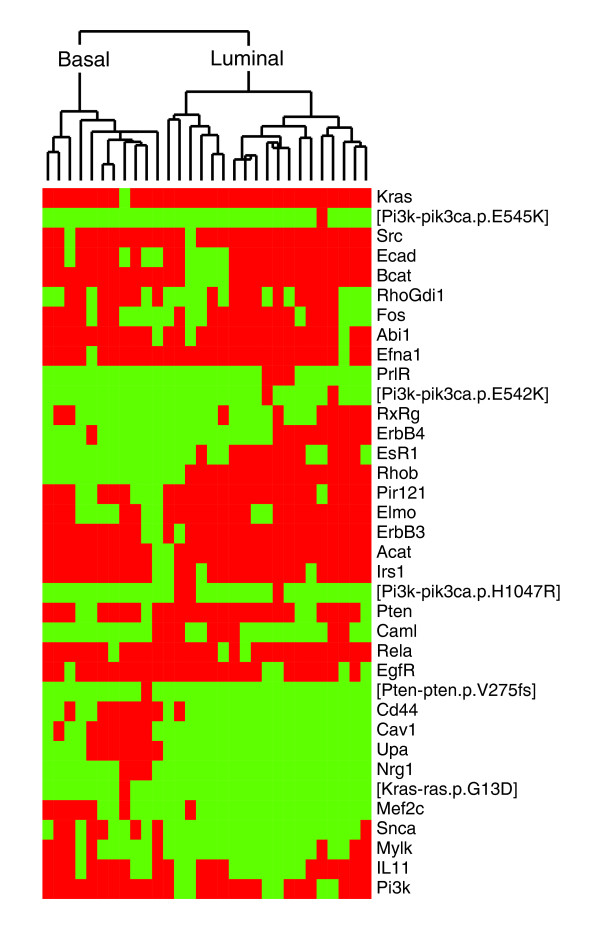
Initial states recapitulate the known biology. Heatmap shows the components in the initial states that varied across the cell lines. Each column represents the initial state from a single cell line network; each row represents data for one component. Red indicates the component is present in the cell line model; green indicates it is absent. Data are hierarchically clustered along both dimensions. Basal and luminal cell lines cluster into distinct groups.

We found that 39 out of 286 (13%) of the components vary across the initial states of the cell lines (Figure 2). This includes both the effect of data discretization, as well as differences in mutational status for Kras, Pten and Pik3ca. The components that vary are located throughout the network and include receptors, GTPases and transcription factors. We used unsupervised hierarchical clustering to analyze the variable components in the initial states [53]. In accordance with our previous studies, we found that the site of origin, basal or luminal epithelium, largely defines the two major clusters [43]. We achieved a similar result when we clustered the data with a partitioning around medoids (PAM) algorithm that searched for two groups in the discretized data. Specifically, most of the cell lines (26 out of 30) correctly segregated into basal or luminal groups. This finding demonstrates that our modeling system has some of the genes that influence this phenotypic difference. Further, it indicates that the discretized data used to populate the network models recapitulate some of the known cell biology associated with the origins of the breast cancer cell lines.

### The network models are highly variable

A principal interest in modeling these pathways was to determine how network topology differs across the set of cell lines. To address this question, we determined which components and rules were present in each of the networks. The network models contain an average of 334 (8.29 standard error of the mean) rules and 218 (4.55 standard error of the mean) unique state changes. Over 55% of the rules and state changes differ across the 30 models, indicating that the networks are highly variable (Table 2). This result was surprising at first, considering that the initial states have 87% of the components in common.

**Table 2:** Summary of network features for the cell line models

	Total	Number variant	Percent variant
Rules	396	248	60
State changes	253	141	55
Initial state	286	39	13

**Figure 3:**
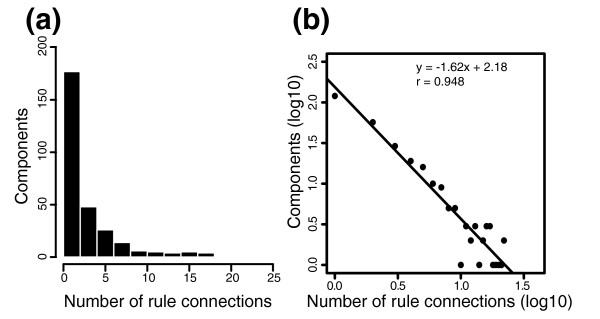
Network connectivity follows a power-law relationship. **(a) **Distribution of the number of rule connections for each component in the model. Most components have only a few rule connections. **(b) **Log-log plot. Each dot represents the number of components in the model that have a particular number of rule connections. The line represents the least-squares fit to the data.

To explore this finding further, we examined the connectivity of individual components by determining the number of rules in which each component is involved. The majority of the components participate in only one or two rules, whereas a few components participate in many rules (Figure 3a). EgfR, the most highly connected component, is involved in 22 rules. When we plotted these data on a log-log plot, a robust linear relationship was revealed, indicating that the connectivity follows a power-law (Figure 3b). Interestingly, some of the most highly connected components vary across the initial states of the cell lines, namely EgfR, Src, Pi3k, and Kras (Table 3). These proteins have a particularly large role in shaping network topology. If they are omitted from the initial state, many rules will fail to fire and many pathways in the resultant network will be truncated.

**Table 3:** The most highly connected components in the network model

Component	Number of rule connections	Variable across initial state
EgfR	22	Yes
Pi3k	20	Yes
Src	18	Yes
Kras	17	Yes
RhoB	17	Yes
RhoA	17	No
Cbl	16	No
Cdc42	16	No
Rac1	16	No
Erk1	15	No
Erk2	15	No
Hras	14	No
Grb2	13	No
PIP2	13	No
Raf1	13	No
Smad2	12	No
Acta1	11	No
EphA2	11	No
Pkca	11	No

**Figure 4:**
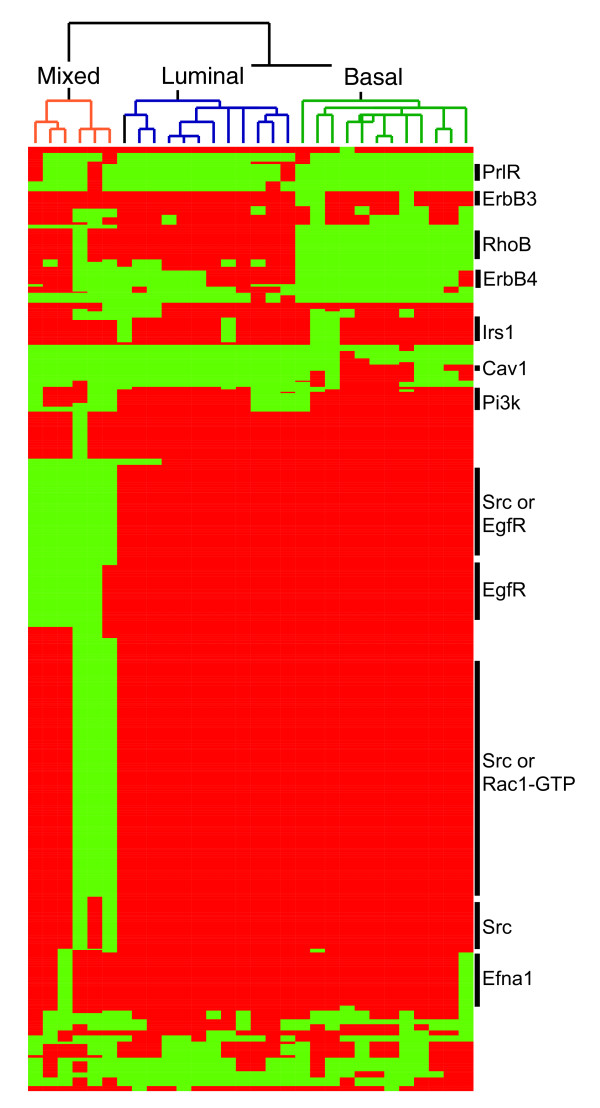
The network models cluster into basal, luminal and mixed groups of cell lines. Heatmap shows the network features that varied across the cell line network models. Each column represents data from one network model; each row represents data for one network feature (component in the initial state, rule or component that underwent a state-change). Red indicates the component is present in the cell line; green indicates it is absent. Hierarchical clustering along the vertical dimension reveals that the networks form basal, luminal and mixed clusters. Hierarchical clustering along the horizontal dimension yields 30 signaling modules, each of which represents a small subnetwork. Signaling modules of particular interest, along with the key components in the initial state, are noted along the right side.

We were interested in whether the cell line models could be grouped by their network properties. We addressed this by performing an unsupervised hierarchical clustering of the network features (that is, the components in the initial state, rules, and components that underwent state changes) that differed across the cell lines. This clustering resulted in three major groups for the cell line models: basal, luminal and a third group composed of both basal and luminal cell lines (Figure 4). The observation that there is a mixed group of basal and luminal networks indicates that the cell lines may be segmented by their signaling pathways, rather than by site of origin alone.

### Unique signaling modules are active in particular subsets of the network models

We next asked how the network structure varies across the cell lines. To answer this question, we used PAM clustering to partition the network features into 30 clusters. Each cluster represents a unique 'signaling module' that is present in some cell line models and absent from others. A summary of these signaling modules provides an overview of the variable network features (Table 4). Each signaling module is driven by the presence of particular components in the initial state. For example, the ErbB4 module is present in ten cell lines, nine of which are luminal and one that is basal, reflecting the fact that ErbB4 is present in the initial state of these ten cell lines. The signaling modules average eight rules each, though they vary in size from a single rule up to 76 rules for the Src/Rac1 module.

**Table 4:** Summary of signaling modules

	Number of rules	Key component(s)	Summary of key events
1	1	Pi3k, ErbB4	ErbB4 activation of Pi3k
2	1	Snca	Pyk2 activation of Snca
3	1	Caml, Rsk	Rsk activation of Caml
4	1	Stat3	Stat3 activation by EgfR
5	1	Irs, Pi3k	Irs activation of Pi3k
6	1	Rela	Formation of Ikba, Nfkb1, Rela complex
7	2	Pik3ca-mut	Akt signaling through Pi3k mutant
8	2	Mef2c	Camk activation of Mef2c
9	2	IL11R, Jak	IL11R activation of Jak
10	2	Elmo, Rac1	Elmo activation of Rac1
11	2	Abi1, Pir121	Wave1 activation dependent on Abi1 and Pir121
12	3	Mylk	Mylk activation of Mlc
13	3	RhoB	RhoB activation
14	3	EsR1, Bcat	EsR1 activation by Rsk; Bcat activation
15	3	Fos	Fos activation by Erk
16	3	Bcat	Activation and degradation of Bcat
17	4	Cav1, UpaR	Integrin/Cav1 activation of Shc; UpaR activation
18	5	Pten, Kras, Pik3ca	Mutation rules
19	5	ErbB4	ErbB4 activation of ErbB2, Shc; Grb2 relocation
20	6	PrlR	PrlR signaling
21	7	Irs1	Irs1 activation; Grb2 translocation
22	8	Pi3k	Eight ways to activate Pi3k
23	12	RhoB	RhoB activation of first-order effectors
24	12	Cbl	Cbl-related signaling, including Rap1a, Crk, Dock
25	14	Src	Src-related signaling, including Fak, Pax, Cas
26	15	EgfR	First-order EgfR interactions including ErbB2, Grb2, Cbl
27	16	Efna1	EphA2/Efna1 signaling; Integrin deactivation by EphA2
28	27	ErbB3	ErbB3 activation by Nrg1 and ErbB2; ErbB3 activation of Shc
29	32	EgfR, Src	Src-dependent activation of EgfR; Cdc42 signaling; activation of Src effectors
30	76	Src, Rac1-GTP	Rac1 signaling; MAPK activation

The RhoB (ras homolog gene family, member B) module is largely responsible for the segmentation of the basal and luminal cell line models, and is present in all the luminals and absent from all the basals. RhoB interacts with NGEF (Ephexin, EPH receptor interacting exchange protein) to activate many downstream targets that go on to regulate a diverse array of cellular functions, including cell motility, cell adhesion and cell cycle progression [54,55]. RhoB levels have been shown to decrease as cancer progresses [56-58]. In accordance with this, we have found that the basal cell lines are far more invasive than the luminal cell lines [43].

Clustering of the 'mixed' group of cell lines is strongly driven by the three Src modules (Figure 4). Src is one of the most highly connected components in the network (18 rules), and serves to integrate a variety of signals. This module, which results from the omission of Src from the initial state, is present in all cell lines except two, basaloid MDAMB435 and luminal MDAMB453. The other two Src modules are dependent on the presence of either EgfR or Rac1. The Src/EgfR module includes Src-dependent activation of EgfR; if either component is missing from the initial state, signaling along this cascade is compromised. The Src/EgfR module is absent only from the mixed group of networks: four are missing EgfR, one is missing Src, and the other is missing both EgfR and Src.

**Figure 5:**
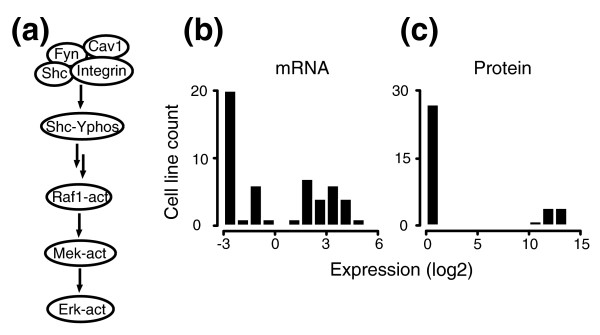
Cav1/Integrin signaling module is present in basal cell lines. **(a) **Signaling module. Cav1, Integrin and Fyn interact to activate SHC, which leads to activation of the MAPK cascade. **(b, c) **Distribution of Cav1 transcript (b) and protein (c) levels across the cell lines. Both datasets show a bimodal distribution of Cav1.

One small signaling module is related to the presence of Cav1 in the initial state. One of the rules in this module describes activation of Shc that is dependent on Fyn (Proto-oncogene tyrosine-protein kinase Fyn), Cav1 and Integrin (ITGB1) (Figure 5a). Both the transcript and protein data indicate that the presence of Cav1 is bimodal, and is clearly present at either very low or very high levels (Figure 5b,c). This module is only present in basal cell lines, and, further, most of the cell lines that contain it are of the most aggressive basal B subtype [43]. This signaling module provides a direct feed into the Raf-Mek-Erk pathway, suggesting that these cell lines have an alternative route available for Erk activation (Figure 5a). This interaction may help to explain why these basal cell lines are particularly aggressive.

### Pak1 plays a pivotal role in the network models

**Figure 6:**
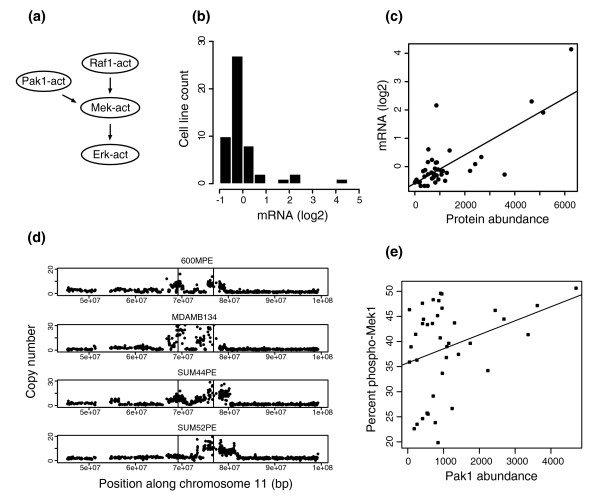
Pak1 is a critical component of the MAPK cascade in our network models. **(a) **Subnet shows that Pak1 leads directly to activation of Raf, Mek and Erk. **(b) **Distribution of Pak1 transcript levels used in construction of the initial states. Pak1 yields two clusters: a lower 'absent' cluster centered at -0.26 and an upper 'present' cluster centered at 2.16. **(c) **Pak1 protein and transcript levels are correlated. Protein abundance is plotted on the x-axis; transcript data (log2 scale) is plotted along the y-axis. The line represents the least-squares fit to the data. **(d) **Copy number profiles for the region around the Pak1 amplicon on chromosome 11. The vertical lines represent the locations of CCND1 (69 Mb) and Pak1 (76 Mb). **(e) **Pak1 protein levels are correlated with percent phospho-Mek1. Each dot represents data from one cell line. The line represents the least-squares fit to the data.

In our model, Pak1 is required for the activation of Mek and Erk (Figure 6a). Specifically, Pak1 phosphorylates Mek, which in turn facilitates signaling along the Raf-Mek-Erk cascade [59]. It follows, then, that network models with Pak1 omitted from the initial state fail to activate Erk. Across the cell lines, the distribution of Pak1 transcript levels is highly skewed, so our discretization algorithm yields two clusters, a large group centered at -0.26, and a small group centered at 2.16 (Figure 6b). Pak1 is present in the initial state of the cell lines with high expression and absent from the others. The four cell lines with high Pak1 transcript levels, MDAMB134, 600MPE, SUM52PE and SUM44PE, are all of luminal origin.

Based on the observations that Pak1 directly regulates MAPK signaling, and that its expression pattern shows substantial variation in breast cancers, we hypothesized that Pak1 differentially regulates MAPK signaling across our panel of cell lines. We tested this hypothesis experimentally. The first issue we addressed was whether Pak1 protein levels vary across the cell lines. We found highly variable expression of total Pak1 protein. Specifically, three of the four cell lines with elevated Pak1 transcript levels have concordantly high Pak1 protein levels. In addition, a handful of other cell lines also show over-expression of Pak1 protein. Pak1 transcript and protein levels are significantly correlated (Pearson's r = 0.78, *P *< 0.0001; Figure 6c). While this relationship is largely dependent on the cell lines that highly express Pak1, it nonetheless supports the idea that elevated transcript levels affect protein expression levels. Focal changes in copy number are thought to convey a selective advantage for tumor growth, so we next asked whether Pak1 is amplified in any of our cell lines. The four cell lines that over-express Pak1 show high-level amplification (>8.7 copies; see Materials and methods) of the Pak1 amplicon (11q13.5-q14 [60]; Figure 6d); none of the other cell lines show this amplification. In addition to Pak1 amplification, three of these cell lines also show amplification at CCND1, though in all cases there are distinct peaks at each locus.

If Pak1 indeed regulates MAPK signaling, we would expect to find a correlation between Pak1 and phospho-Mek levels. To address this, we quantified isoform-specific phospho-Mek levels in our cell lines (see Materials and methods). We found a small but significant correlation between total Pak1 and percent Mek1-S298 (Pearson's r = 0.32, *P *< 0.05; Figure 6e). Although the correlation is somewhat weak, it is clear that high Pak1 levels are always associated with elevated phospho-Mek1. In accordance with the observation that the interaction between Pak1 and Mek is specific to Mek1 [61], we found no correlation between Pak1 and percent phospho-Mek2 (*P*>> 0.05).

**Figure 7:**
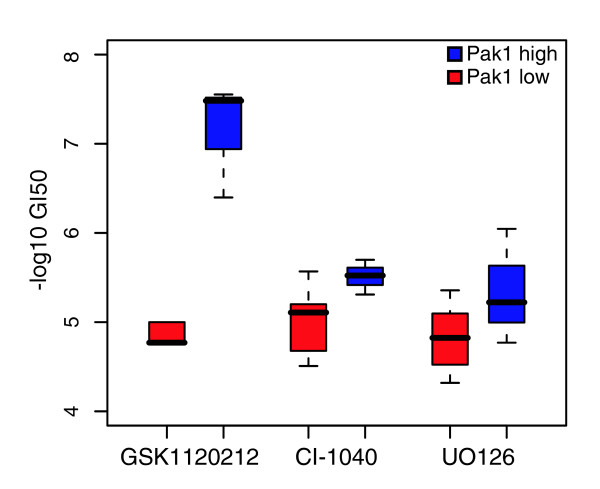
Pak1 over-expression predicts responsiveness to Mek inhibitors. Each pair of boxplots represents the average GI_50 _for luminal cell lines that over-express Pak1 (Pak1-high, blue) and those that express it at normal levels (Pak1-low, red). Within each box, the line represents the median; upper and lower boundaries represent the first and third quartiles, respectively. The vertical lines extend to +/- 1.5 IQR. For all three drugs, Pak1-high cell lines are significantly more sensitive than Pak1-low cell lines.

The above findings suggest that elevated Pak1 levels provide a foothold into regulation of the MAPK cascade, and led us to hypothesize that Pak1 over-expressing luminal cell lines would be particularly sensitive to Mek inhibition. To test this, we measured the response of 20 luminal cell lines to three Mek inhibitors: CI-1040, UO126 and GSK1120212. We compared growth inhibition (GI_50_, the drug concentration required to inhibit growth by 50%) following drug exposure between cell lines that over-express Pak1 (n = 3) and those that do not (n = 17). The two groups of cell lines had significantly different mean expression of both the Pak1 transcript and protein (*t*-test, *P *< 0.01). The three Pak1 over-expressing cell lines (MDAMB134, SUM52PE and 600MPE) were significantly more sensitive to Mek inhibition compared to the non-Pak1 over-expressing cell lines (GSK1120212, *P *< 0.005; CI-1040, *P *< 0.05; UO126, *P *< 0.05; *t*-test; Figure 7). This result indicates that Pak1 over-expression may be a useful clinical marker to determine whether a particular tumor will be responsive to Mek inhibition.

## Discussion

Cancer arises from deregulation in any of a multitude of genes, but exactly how this deregulation impacts cell signaling is not well understood. Here, we leveraged a rich dataset of transcriptional and protein profiles with a computational modeling system in order to gain a greater understanding of the critical signaling pathways associated with breast cancer. By creating a unique network model for individual cell lines, we were able to identify signaling pathways that are particularly important in subsets of the cell lines. Our modeling led to new insight about the importance of Pak1 as a modulator of the MAPK cascade.

### Approaches to computational modeling

There are many approaches to computationally modeling biological systems, ranging from high-level statistical models to low-level kinetic models [62]. We used a simplified mid-level scheme to construct network models from transcript and protein profiles for two reasons. First, we were able to create a unique model for each cell line, rather than a single network that represents 'breast cancer.' We used this approach to examine how a collection of genomic and proteomic changes in individual cell lines affects its network architecture. In contrast, other approaches, such as Bayesian reconstruction, are designed to describe ensemble behavior, rather than behavior of individual cell lines [63,64]. A key attribute of our modeling system is that it can be used to identify specific biological instances of cell signaling that can be used to generate hypotheses. Our observations about Pak1 are a key example of this feature. The second reason for using this mid-level modeling scheme is that the computational algorithm is relatively simple; logical operators define relationships between signaling components. It is therefore possible to create networks that are quite large, which provides the opportunity to examine multiple inputs that impinge upon the central signaling pathway of interest. In comparison, kinetic models that offer more detail about signaling components are quite computationally demanding, so it is only feasible to examine a limited number of components [65,66]. As a 'hypothesis generator,' our modeling system could be used to guide the development of dynamic modeling systems by identifying key signaling components to include in them.

One limitation of our modeling system is that it operates in a totally discrete manner: components are either present or absent, and rules fire with absolute certainty or not at all. This is a simplification of true biological systems in which the levels of signaling components show a wide dynamic range, and the probability that a reaction will occur changes as a function of the concentration of individual proteins. We captured the variation in the concentration of signaling components by individually discretizing the data for each component in the initial state and then assigning each cell line to a 'present' or 'absent' group. With this approach, we examined how signaling is affected by extreme changes in protein levels, therefore homing in on key signaling events. We found that even with this simplified approach, we were able to make insights into key signaling events in subsets of our cell lines. Hybrid modeling approaches, which combine continuous dynamical systems with discrete transition systems, have been developed to overcome this limitation [67,68]. Modification of the current model system to a hybrid system would allow for a more detailed examination of cell signaling over smaller changes in protein concentrations.

### Modeling results

We found that the network connectivity follows a power law relationship in which most components have low connectivity while a few components are highly connected (Figure 3). The relationship we observed reflects not only intrinsic connectivity, but also curation bias, as literature relevant to EgfR/MAPK signaling was preferentially surveyed during creation of the rule set. Nonetheless, this 'scale free' relationship has been described in more thorough surveys of protein-protein interactions [69,70]. The observation that our network models have this scale free property supports the idea that they are biologically relevant representations. Further, this pattern of connectivity implies that the few highly connected components may be most critical for regulating cell signaling along these pathways - these components serve as promising candidates for more detailed study at both the computational and experimental levels. Those that also show substantial variation across the cell lines (for example, EgfR, Src, Pi3k, and Kras) may be particularly relevant in the context of breast cancer.

Traditionally, the site of origin has been one of the primary features with which to classify breast cancers [23-25]. The full transcriptional profiles of our cell line panel show this characteristic split between basal and luminal subtypes [43], which we could largely recapitulate in our construction of the initial states (Figure 2). Here, we have shown that ErbB/MAPK signaling systematically varies across our panel of cell lines. Specifically, we found that the cell line networks could be classified into three groups (Figure 4). The basal and luminal network groups reflect the split we observed in the components of the initial state, while the third mixed group is largely defined by signaling related to Src. Src acts as a well-connected signaling hub, so it is particularly important in shaping network architecture. It also interacts with several key proteins in the MAPK cascade, including EgfR and its targets, Erk, and Cdc42 [71,72]. Src has been studied as a therapeutic target in a wide range of cancers, including cancers of the breast, lung and pancreas [73,74].

The basal and luminal networks could be well-differentiated by the RhoB signaling module, which is present in the luminal cell lines and absent from the more aggressive basal cell lines (Figure 4). A number of reports have indicated that loss of RhoB expression is frequently associated with cancer progression [58]. Furthermore, suppression of RhoB is a critical step leading to transformation in a variety of cancers, including those of the lung and cervix [75]. These observations bolster the idea that modulation of the RhoB pathway may serve as a useful therapy in the basal cell lines. Among the basal cell line networks, the Cav1/Integrin signaling module was primarily found in the most aggressive basal B cell lines. In accordance with this, Cav1 has been shown to have a role in carcinogenesis, though its mechanism may vary with cancer type [76,77].

### Pak1 impacts signaling along the MAPK cascade

Through an analysis of our breast cancer network models, we identified Pak1 as a putative differential regulator of the MAPK cascade in our cell lines. Pak1, a serine/threonine kinase, has long been studied as a regulator of cytoskeletal remodeling and cell motility [78,79], but more recently has been shown to regulate both proliferation [80] and apoptosis [81]. The Pak family of proteins has been implicated in a variety of cancers, including those of the breast [80,82,83]. In particular, Pak1 hyperactivation has been shown to cause mammary-gland tumors in mice [84].

Across our panel of cell lines, Pak1 is differentially expressed at the copy number, transcript and protein levels (Figure 6). The finding of elevated Pak1 expression in some of our cell lines mirrors the observation that Pak1 is sometimes upregulated in breast tumors [80]. The correlation between Pak1 and phospho-Mek1 levels (Figure 6c) suggests that across the cell lines, Pak1 differentially modulates activation of the MAPK cascade. Although statistically significant, this correlation was not perfect: high Pak1 levels are always associated with high phospho-Mek1 levels, while a more variable relationship emerges when Pak1 is low. This observation implies that when Pak1 levels are high, it dominates the regulation of phospho-Mek1, whereas at low Pak1 levels, alternate proteins must serve as the principle regulator of phospho-Mek1. For example, Ksr1 (Kinase suppressor of ras-1) and Spry (sprouty homolog, antagonist of FGF signaling) are both involved in regulation of the MAPK cascade, and may be particularly important in the cell lines that express Pak1 at low levels [85,86]. Based on this finding, we hypothesized that the luminal cell lines that over-express Pak1 would be particularly sensitive to Mek inhibition. Indeed, the Pak1 over-expressing cell lines were significantly more sensitive to three Mek inhibitors than the non-Pak1 over-expressing cell lines (Figure 7). The observation that all three drugs showed the same pattern indicates that the inhibition is quite robust and not due to off-target effects. These results indicate that Pak1 over-expression may be a useful clinical marker to determine which patient populations may be sensitive to Mek inhibitors.

## Conclusions

Breast cancer is a remarkably heterogeneous disease that results from the accumulation of various genetic defects. We were interested in identifying signaling subnetworks that may be particularly important in generating oncogenic phenotypes. To address this, we generated a discrete, static network model for a panel of 30 breast cancer cell lines. The resultant network models were highly variable: of the protein interactions predicted to occur, over half of them varied across the cell lines. We searched for active subnetworks by clustering the network features of our models. This clustering yielded three main groups of cell lines, a basal group, a luminal group, and a third mixed group composed of both basal and luminal cell lines. In addition, we identified several network modules active in specific subsets of the cell lines. One signaling module implicated Pak1 as a key regulator of the Raf-Mek-Erk pathway in the cell lines that over-express it. Based on this observation, we hypothesized that luminal cell lines that over-express Pak1 would be particularly responsive to Mek inhibition. In support of this idea, we found that among luminal cell lines, the over-expression of Pak1 was indeed significantly associated with sensitivity to three Mek inhibitors. All together, these results indicate the utility of symbolic systems modeling for the identification of key cell signaling events in the context of cancer.

## Materials and methods

### Cell lines

The complete panel contains 51 breast cancer cell lines that have been previously described [43]. We assembled our panel of breast cancer cell lines from the ATCC and the laboratories of Drs Steve Ethier and Adi Gazdar. All cell lines have been carefully maintained in culture, and we have stored stocks of the earliest-passage cells. We assure quality control by careful analysis of morphology, growth rates, gene expression and protein levels over time. All extracts were made from subconfluent cells in the exponential phase of growth in full media. Information about biological characteristics and culture conditions is available elsewhere [87]. We generated network models for the 30 well-characterized cell lines with the complete datasets described below.

### Protein abundance data

We measured the abundance of 25 proteins associated with ErbB/MAPK signaling in our network model. These abundances were assayed and quantified as previously described [43]. Briefly, proteins were measured by western blots of cells lysed in 1% Nonidet-P40, 50 mM HEPES (pH 7.5), 150 mM NaCl, 25 mM b-glycerophosphate, 25 mM NaF, 5 mM EGTA, 1 mM EDTA, 15 mM pyrophosphate, 2 mM sodium orthovanadate, 10 mM sodium molybdate, leupeptin (10 mg/ml), aprotinin (10 mg/ml), and 1 mM phenylmethylsulphonyl fluoride (PMSF).

We quantified protein levels by measuring the emitted chemiluminescence or infrared radiation recorded from labeled antibodies using Scion Image [88] or Odyssey software [89]. For each protein, the blots were made for 4 sets of 11 cell lines, where each set included the same pair (SKBR3 and MCF12A) to permit intensity normalization across sets. We performed a basic multiplicative normalization by fitting a linear mixed-effects model to log intensity values, and adjusted within each set to equalize the log intensities of the pair of reference cell lines across the sets.

### Transcriptional profiles

Total RNA was prepared from samples using Trizol reagent (GIBCO BRL Life Technologies; Carlsbad, CA, USA) and quality was assessed on the Agilent Bioanalyser 2100. Preparation of *in vitro *transcription products, oligonucleotide array hybridization, and scanning were performed according to Affymetrix (Santa Clara, CA, USA) protocols. In brief, 5 μg of total RNA from each breast cancer cell line and T7-linked oligo-dT primers were used for first-strand cDNA synthesis. *In vitro *transcription reactions were performed to generate biotinylated cRNA targets, which were chemically fragmented at 95°C for 35 minutes. Fragmented biotinylated cRNA (10 μg) was hybridized at 45°C for 16 h to an Affymetrix high-density oligonucleotide array human HG-U133A chip. The arrays were washed and stained with streptavidin-phycoerythrin (final concentration 10 μg/ml). Signal amplification was performed using a biotinylated anti-streptavidin antibody. The array was scanned according to the manufacturer's instructions (2001 Affymetrix Genechip Technical Manual). Scanned images were inspected for the presence of obvious defects (artifacts or scratches) on the array. Defective chips were excluded, and the sample was reanalyzed.

We generated probe set based gene expression measurements from quantified Affymetrix image files with the RMA algorithm [90] from the BioConductor tools suite [91] and annotated with Unigene annotations from the July 2003 mapping of the human genome [92]. All 51 CEL files were analyzed simultaneously, yielding a data matrix of probe sets by cell lines in which each value is the calculated log abundance of each gene probe set for each cell line. Gene expression values were centered by subtracting the mean value of each probe set across the cell line set from each measured value.

### Mutation data

We searched the Sanger Catalogue Of Somatic Mutations In Cancer (COSMIC) website for reported mutations in our cell lines [46]. We incorporated mutations to Kras, Pten and Pik3ca into our models through the construction of rules that reflect the functional impact of each mutation.

### Copy number profiles

We measured copy number profiles with molecular inversion probes (MIPs). The MIP assay was performed as previously described [93]. Briefly, test DNA samples were diluted to 16 ng/ml. All DNA quantification was done using PicoGreen dsDNA Assay Kit (P7589; Molecular Probes/Invitrogen, Carlsbad, CA, USA). We used 96- or 384-well plates whenever possible to reduce variation. For day 1 overnight annealing, 4.7 μl of DNA samples (75 ng total), 0.75 μl of Buffer A, 1.1 μl of the 53 K probe pool (200 amol/μl/probe) and 0.045 μl of Enzyme A were mixed well in a 384-well plate on ice. The reaction was incubated at 20°C for 4 minutes, 95°C for 5 minutes, then 58°C overnight. On day 2, 13 μl of Buffer A was added to each well with 1.25 μl of Gapfill Enzyme mix, then 9 μl of this was put in each of two wells in a 96-well plate. MIP probes were circularized with 4 μl of dinucleotide (dATP with dTTP, dCTP with dGTP) and mixed at 58°C for 10 minutes. The uncircularized probes and genomic DNA were eliminated by addition of 4 μl of Exonuclease Mix and incubation at 37°C for 15 minutes, followed by heat-killing of enzymes. The circularized probes were linearized by the addition of Cleavage Enzyme Mix at 37°C for 15 minutes, then subjected to universal primer amplification for 18 cycles at 95°C for 20 s, 64°C for 40 s, and 72°C for 10 s. For the labeling reaction, the product was further amplified with the label primers for 10 cycles, and then subjected to cleavage by Digest Enzyme Mix at 37°C for 2 h. To hybridize, the cleaved MIP products were mixed with hybridization cocktail, denatured and hybridized to 70 K Universal Taq arrays at 39°C for 16 h (two arrays per sample). The overnight hybridized arrays were washed on GeneChip^® ^Fluidics Station FS450 and stained by streptavidin-phycoerythrin at 5 ng/ml (Invitrogen). Copy number estimation was obtained from the hybridization signals as previously described [93].

We filtered the dataset to eliminate MIP probes missing from more than 5% of the samples. We used the previously described amplicon boundaries to compute average copy number across all the probes in the Pak1 and CCND1 amplicons [60]. We defined high-level amplification as Median copy number + (3 × Interquartile range), each computed across all amplicons and cell lines.

### Quantitative analysis of Mek

We used high-resolution capillary isoelectric focusing technology to quantify the abundance of individual phosphoforms and isoforms of Mek. We used Mek1 (Upstate Biotechnology, Lake Placid, NY, USA) and Mek2 (Cell Signaling, Danvers, MA, USA) antibodies for this assay, which has been described in detail elsewhere [94].

### Cell growth inhibition assay and data analysis

Cells were plated at proper density in 96-well plates such that they would remain in log growth at the end of assay time. The cells were allowed to attach overnight before being exposed to Mek inhibitor CI-1040, UO126 or GSK1120212 for 72 h. Drugs were dissolved in dimethyl sulfoxide (DMSO) as 10 mM stock, and a set of 9 doses in 1:5 serial dilution was added in triplicate wells. The final DMSO concentration in the treated well was 0.3% or less. The cell growth was determined using Cell Titer Glo assay (CellTiter-Glo Luminescent Cell Viability Assay; Promega, Madison, WI, USA), with slight modification from the manufacturer's protocol at day 0 (time when drug was added) and day 3 of drug exposure. Briefly, Cell Titer Glo reagent was diluted with phosphate-buffered saline (1:1 v:v) and the culture media was removed from the 96-well plate prior to adding 50 μl per well of the diluted Cell Titer Glo reagent. Luminescence from the assay was recorded using BIO-TEK FLx800.

Data calculations were made according to the method described by the NCI/NIH DTP Human Tumor Cell Line Screen Process [95] and as previously described [96]. The percent growth curve is calculated as [(T - T_0_)/(C - T_0_)] × 100, where T_0 _is the cell count at day 0, C is the vehicle control (for example 0.3% DMSO without drug) cell count at day 3, and T is the cell count at the test concentration. We calculate the GI_50 _and total growth inhibition (TGI) values after 72 h drug exposure. The GI_50 _is the drug concentration that results in 50% growth inhibition; the TGI is the drug concentration that yields 100% growth inhibition.

### Pathway Logic modeling system

Pathway Logic [97] is a system for building discrete, logical models of biological systems [35,36]. The construction of a Pathway Logic model requires two key elements: a set of rules and an initial state. Each rule represents a statement of a precisely defined biological transformation or biochemical reaction. For example, the rule below describes the activation of the ErbB2 receptor by activated EgfR:

rl[793.ErbB2.on]:

{CLm | clm [EgfR - act] ErbB2}

= >

{CLm | clm [EgfR - act] [ErbB2 - act]}.

The first term on each line represents a cellular location. In this case, CLm indicates that EgfR and ErbB2 are located in the cell membrane. A reaction will occur ('fire') only if the components are located in the specified cellular compartment. Most rules in our database describe changes to the state of a protein, such as activation, exchange of GDP for GTP, or translocation to a different cellular compartment. In total, the relevant rule database contains 396 rules, all of which have been individually curated from primary literature sources.

The initial state specifies the model components present in a cell, as well as their locations. We created the initial states for each network model from a set of 286 components. Models are generated by 'rewrites.' In a simple rewrite, the initial state is presented to the rules. Whenever the state meets the conditions required by a rule input, the state is adjusted in accordance with the rule. The new state is then presented to the rules and more adjustments are made. This iterative process continues until either no further alterations can be made, or a user-defined condition is reached. We visualize the result of these rewrites as a Petri net, a directed bipartite graph that contains places, transitions, and directed arcs that connect the places and transitions [98]. In Petri net models of cell signaling, places represent proteins and transitions represent chemical reactions. Petri nets are a useful representation because they closely resemble hand-drawn cartoon models of cellular signaling pathways.

### Data discretization

We discretized the protein and transcript data in order to determine which components were present in (or absent from) the initial state of each cell line network model. Conceptually, the idea was to analyze the expression data for each protein in the initial state in order to decide if it showed differential expression across the panel of cell lines. Proteins that showed a highly variable expression pattern across the panel of cell lines were considered present in some cell lines and absent from others. Our approach to discretization and creation of the initial states was quite conservative. That is, we did not omit a component from the initial state unless there was strong evidence that it is absent from a particular cell line. We chose a conservative approach because in discrete networks such as these, errant omission of a component from the initial state can lead to significant effects on the structure of the network, in the form of truncated signaling pathways (Figure 1b).

We developed the following discretization method and applied it to both the protein and transcript data. First, for each gene or protein, we used PAM clustering and a mean split silhouette (MSS) statistic to determine whether the log-transformed expression values are best represented as 1, 2 or 3 groups of cell lines [99]. We searched for one, two or three groups because the distributions of expression values appear unimodal (that is, one group; Figure 1c), bimodal (that is, two groups; Figure 1d), or tri-modal (Figure 1e). We used the MSS statistic for three reasons: first, it can be used to classify the expression values as a single group, whereas most algorithms (for example, k-means) require a minimum of two groups; second, it accurately classified both one-tailed and two-tailed distributions; and finally, because it could identify small clusters in the data.

Next, for genes that clustered into two or three groups, we compared the mean expression levels of the groups. If the expression levels between the highest and lowest group differed by less than a four-fold change, we collapsed the groups together. This ensured that expression differences between the groups were great enough to be meaningful. We assigned proteins to the initial states in the following way. If a single group best described the distribution of expression values, the protein was considered present in all the cell lines. For distributions that yielded more than one group, the protein was considered absent from the initial state of the cell lines with the lowest mean expression; the protein was present in the initial state of cell lines in the highest group(s). We considered the protein present in the two clusters with highest mean expression in order to avoid erroneous omissions from the initial state of cell lines in the middle expression group. Finally, if we had no data available from which to estimate the initial state, we considered the protein present in all cell lines.

For model components that had both transcript and protein data available, we used the clustered protein (rather than transcript) data to populate the model. To ensure that we made the most robust initial state assignments possible, we used data from as many of the 51 cell lines for the discretization step, even if we ultimately did not create a network model for the cell line. We performed the analyses above in R with the hopach package, available as part of the BioConductor tools suite [100].

### Analysis of network topology

We used the following method to compare the networks. First, we decomposed each network into a list of all the components and rules contained within it. This list describes all the state changes (for example, phosphorylation) and reactions in each network. We clustered the network features with PAM and an MSS, which searched for the optimal number of clusters, up to a maximum of 40. Each cluster can be considered a unique 'signaling module' that represents a small portion of the total network. We compared the presence or absence of these signaling modules across the panel of cell lines.

### Hierarchical clustering and data visualization

The discretized data used to populate the initial states were hierarchically clustered using an average linkage algorithm and a Pearson correlation for the distance measure [53]. We also used this algorithm to cluster the cell line network models. We used Java TreeView to visualize the clustered data in Figures 2 and 4[101].

## Abbreviations

Cav1: Caveolin-1; DMSO: dimethyl sulfoxide; EgfR: epidermal growth factor receptor; ER: estrogen receptor/EsR1; MAPK: mitogen-activated protein kinase; MIP: molecular inversion probe; MSS: mean split silhouette statistic; PAM: partitioning around medoids; Pi3k: phospho-inositide-3-kinase.

## Authors' contributions

LMH participated in the design of the study, constructed the computational models and drafted the manuscript. NJW conducted the Pak1 molecular studies. CLT created the Pathway Logic software and provided guidance of the computational modeling. MK curated the rule library. MK and KRL provided biological guidance of the modeling work. YG, ZH, SZ and WLK conducted the growth inhibition assays. BLW, SL, JRJ and RFW provided the Mek inhibitor GSK1120212. JWG and PTS conceived of the study and participated in its design and coordination.
